# Supplementary material for: Roles of Candida albicans Mig1 and Mig2 in glucose repression, pathogenicity traits, and SNF1 essentiality
Source: PLoS Genet. 2020 Jan 21;16(1):e1008582. doi: 10.1371/journal.pgen.1008582 (PMC6994163; doi:10.1371/journal.pgen.1008582)
Supplement: S3 Fig — A. Western blots are from biological triplicates used to quantify Mig1-HA tagged protein levels. Protein extracts from strains KL1026 and CW542 were made from overnight cultures washed in H2O, inoculated at OD 0.2, and grown in 50 ml cultures of the indicated medium for 4 h at 37°C. For Spider + Glucose medium, 5 ml of 20% glucose was added to a culture grown in Spider medium at 3 h and 50 min. CW542 protein extracts were used as a non-tagged negative control. Ponceau S was used as a control for proper protein transfer. Samples were visualized with an antibody against HA and membranes were stripped and reprobed with an antibody against Tubulin for loading control. B. Functional phenotyping of Mig1 protein in the Mig1-HA strain using a caspofungin sensitivity plate dilution assay from Fig 6A. The Mig1-HA strain is not more sensitive to caspofungin compared to the wild-type. (PDF) [file pgen.1008582.s003.pdf]

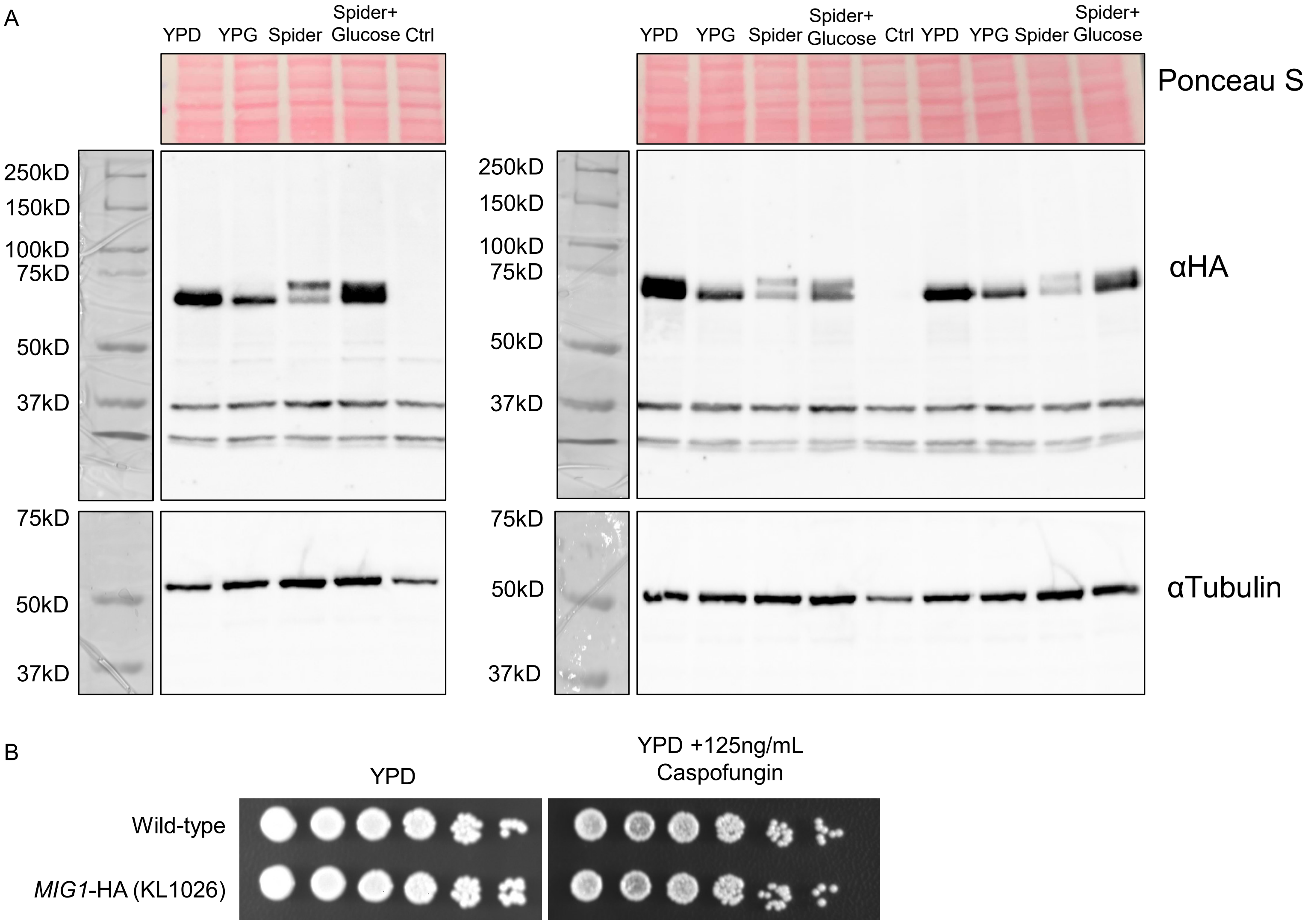

Figure S3. Western blot of Mig1-HA tagged protein grown in media with various carbon sources.

A. Western blots are from biological triplicates used to quantify Mig1-HA tagged protein levels. Protein extracts from strains KL1026 and CW542 were made from overnight cultures washed in H_2O, inoculated at OD 0.2, and grown in 50 ml cultures of the indicated medium for 4 h at 37°C. For Spider + Glucose medium, 5 ml of 20% glucose was added to a culture grown in Spider medium at 3 h and 50 min. CW542 protein extracts were used as a non-tagged negative control. Ponceau S was used as a control for proper protein transfer. Samples were visualized with an antibody against HA and membranes were stripped and reprobed with an antibody against Tubulin for loading control.

B. Functional phenotyping of Mig1 protein in the Mig1-HA strain using a caspofungin sensitivity plate dilution assay from Figure 6A. The Mig1-HA strain is not more sensitive to caspofungin compared to the wild-type.
