# Supplementary material for: Roles of Candida albicans Mig1 and Mig2 in glucose repression, pathogenicity traits, and SNF1 essentiality
Source: PLoS Genet. 2020 Jan 21;16(1):e1008582. doi: 10.1371/journal.pgen.1008582 (PMC6994163; doi:10.1371/journal.pgen.1008582)
Supplement: S4 Fig — Strains: Wild-type (CW542), sak1Δ/Δ (KL988), sak1Δ/Δ mig1Δ/Δ (KL951 and KL952), sak1Δ/Δ mig1Δ/Δ mig2Δ/Δ (KL955) and sak1Δ/Δ mig2Δ/Δ (KL960 and KL962) were grown overnight in YPD and tenfold serial dilutions of the indicated strains were spotted on YPD and Spider plates. Growth was visualized after 2 days of incubation at 37°C. (PDF) [file pgen.1008582.s004.pdf]

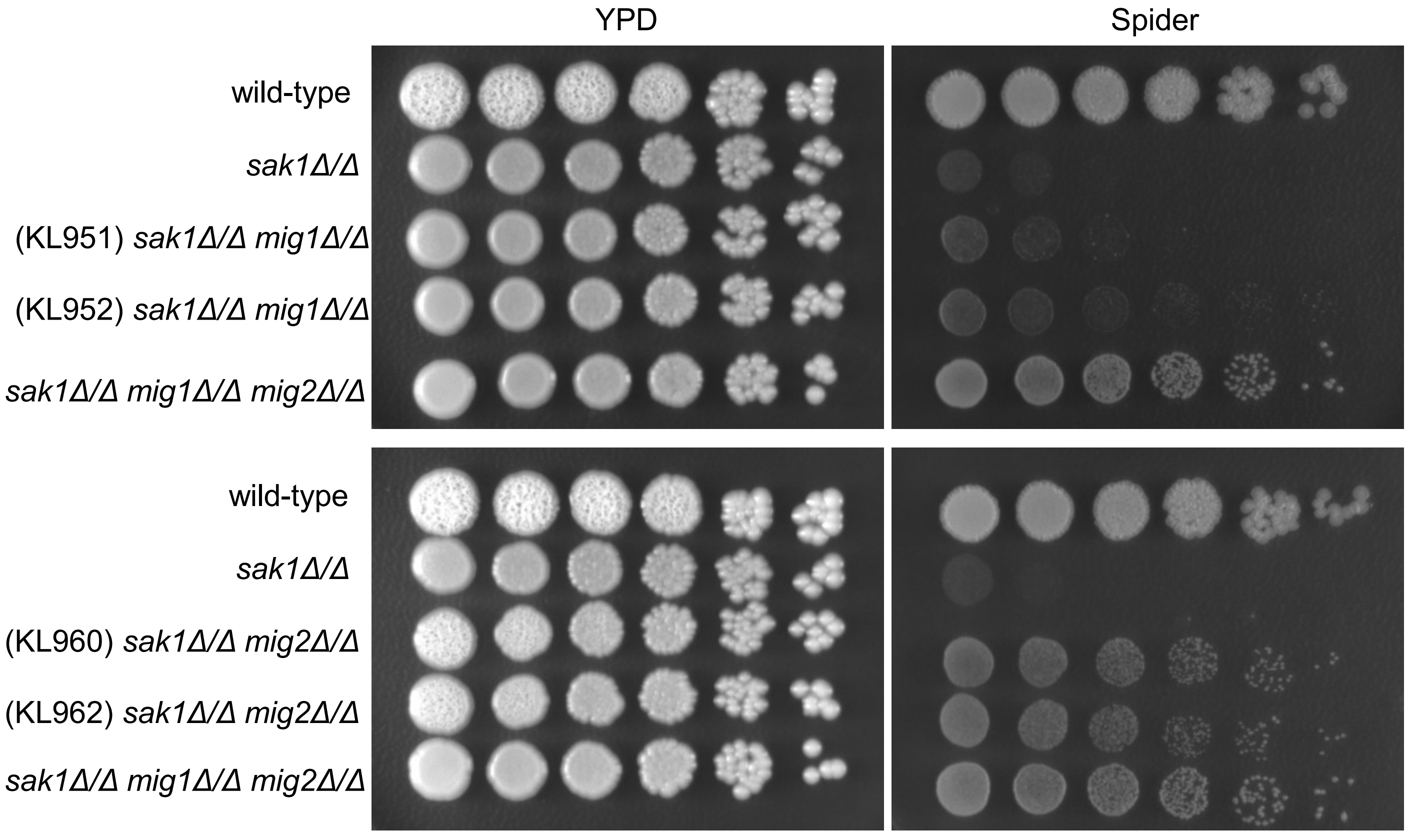

Figure S4. Independent isolates of *sak1Δ/Δ mig1Δ/Δ* and *sak1Δ/Δ mig2Δ/Δ* strains show similar growth phenotypes on Spider media.
Strains: Wild-type (CW542), *sak1Δ/Δ (*KL988), *sak1Δ/Δ mig1Δ/Δ* (KL951 and KL952), *sak1Δ/Δ mig1Δ/Δ mig2Δ/Δ* (KL955) and *sak1Δ/Δ mig2Δ/Δ* (KL960 and KL962) were grown overnight in YPD and tenfold serial dilutions of the indicated strains were spotted on YPD and Spider plates. Growth was visualized after 2 days of incubation at 37° C.
