# Supplementary material for: Roles of Candida albicans Mig1 and Mig2 in glucose repression, pathogenicity traits, and SNF1 essentiality
Source: PLoS Genet. 2020 Jan 21;16(1):e1008582. doi: 10.1371/journal.pgen.1008582 (PMC6994163; doi:10.1371/journal.pgen.1008582)
Supplement: S5 Fig — Strains: Wild-type (CW542), snf1Δ/Δ mig1Δ/Δ (KL953 and KL954), snf1Δ/Δ mig1Δ/Δ mig2Δ/Δ (KL957 and KL958) and snf1Δ/Δ mig1Δ/Δ mig2Δ/Δ + SNF1 (KL974) were spotted at an OD of 0.1 on YPD media and grown at 30°C for 7 days. (PDF) [file pgen.1008582.s005.pdf]

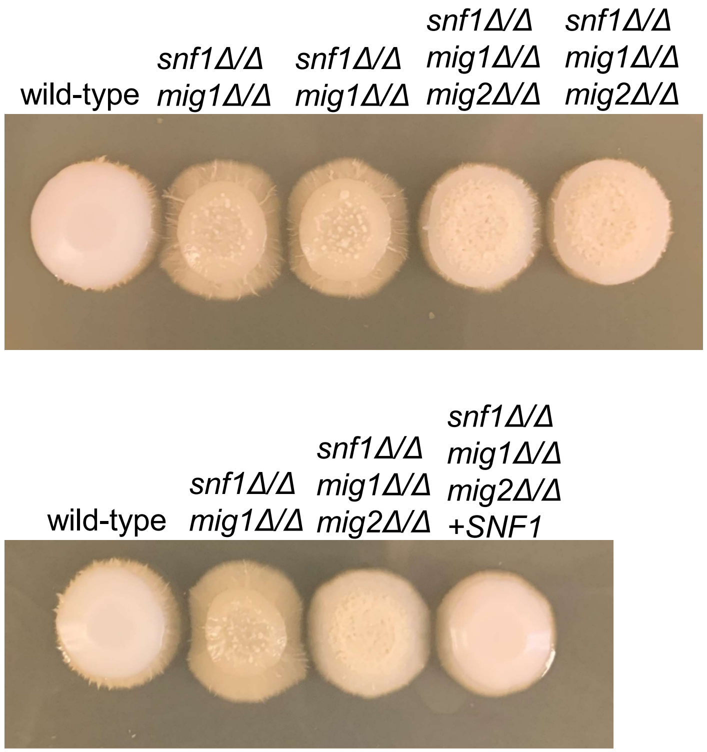

Figure S5. The *snf1Δ/Δ mig1Δ/Δ* strain shows abnormal filamentation and coloration compared to wild-type and the *snf1Δ/Δ mig1Δ/Δ mig2Δ/Δ* strain. Strains: Wild-type (CW542), *snf1Δ/Δ mig1Δ/Δ (*KL953 and KL954), *snf1Δ/Δ mig1Δ/Δ mig2Δ/Δ* (KL957 and KL958) and *snf1Δ/Δ mig1Δ/Δ mig2Δ/Δ* + *SNF1* (KL974) were spotted at an OD of 0.1 on YPD media and grown at 30°C for 7 days.
