# Supplementary material for: Roles of Candida albicans Mig1 and Mig2 in glucose repression, pathogenicity traits, and SNF1 essentiality
Source: PLoS Genet. 2020 Jan 21;16(1):e1008582. doi: 10.1371/journal.pgen.1008582 (PMC6994163; doi:10.1371/journal.pgen.1008582)
Supplement: S1 Text — Details of strain constructions are provided. (DOCX) [file pgen.1008582.s006.docx]

**Strain Construction:**

Strain CW542 (wild-type): One copy of *C. albicans* *ARG4* amplified from SC5314 genomic DNA was integrated at the *arg4Δ* locus of strain SN250. Transformants were selected on CSM media lacking Arginine.

Strain KL820 (*mig1Δ/Δ*): One copy of *C. albicans* *ARG4* amplified from SC5314 genomic DNA was integrated at the *arg4Δ* locus of the *mig1Δ/Δ* strain (A11 X2) from the Homann Deletion Collection [1]. Transformants were selected on CSM media lacking Arginine.

Strain KL738 (*mig2Δ/Δ*) was constructed using SN250 as the parent strain. Transformation mix contained a *MIG2* sgRNA expression cassette created using primers KL1 and KL2, Cas9, and an *ARG4* deletion cassette amplified from pSN69 using primers KL3 and KL4 which contain approximately 80 basepairs of homology upstream or downstream of the *MIG2* coding region. Transformants were selected on CSM media lacking Arginine.

Strain KL742 (*mig1Δ/Δ mig2Δ/Δ*) was constructed using the *mig1Δ/Δ* strain (A11 X2) from the Homann Deletion Collection [1]. Transformation mix contained a *MIG2* sgRNA expression cassette created using primers KL1 and KL2, Cas9, and an *ARG4* deletion cassette amplified from pSN69 using primers KL3 and KL4 which contain approximately 80 basepairs of homology upstream or downstream of the *MIG2* coding region. Transformants were selected on CSM media lacking Arginine.

For *MIG2* complementation, the plasmid pAG6 [2] was cut using the restriction enzymes XhoI and ApaI. Primers KL5 and KL6 were used to PCR the *MIG2* coding region plus 256 basepairs upstream and 536 basepairs downstream of the open reading frame from SC5314 genomic DNA. The cut insert was ligated into pAG6.

Strain KL822 (*mig2Δ/Δ + pMIG2*) was constructed using KL738 as the parent strain. The *MIG2* complementing plasmid was cut with I-SceI for integration at the *RPS1* locus. Transformants were selected for drug resistance on YPD media + nourseothricin.

Strain KL807 (*mig1Δ/Δ mig2Δ/Δ + pMIG2*) was constructed using KL742 as the parent strain. The *MIG2* complementing plasmid was cut with I-SceI for integration at the *RPS1* locus. Transformants were selected for drug resistance on YPD media + nourseothricin.

For *MIG1* complementation, the plasmid pAG6 [2] was cut using the restriction enzymes XhoI and ApaI. Primers KL7 and KL8 were used to PCR the *MIG1* coding region plus 1,088 basepairs upstream and 633 basepairs downstream of the open reading frame from SC5314 genomic DNA. The cut insert was ligated into pAG6.

Strain KL831 (*mig1Δ/Δ + pMIG1*) was constructed using KL820 as the parent strain. The *MIG1* complementing plasmid was cut with I-SceI for integration at the *RPS1* locus. Transformants were selected for drug resistance on YPD media + nourseothricin.

Strain KL830 (*mig1Δ/Δ mig2Δ/Δ + pMIG1*) was constructed using KL742 as the parent strain. The *MIG1* complementing plasmid was cut with I-SceI for integration at the *RPS1* locus. Transformants were selected for drug resistance on YPD media + nourseothricin.

Strain KL938 (*mig1Δ/Δ mig2Δ/Δ + pMIG1*): For validation of the *mig1Δ/Δ mig2Δ/Δ* (KL742) hyphal strain defect one copy of the *MIG1* allele was integrated at the *mig1Δ* locus [3]. Primers KL9 and KL10 were used to PCR the *MIG1* coding region from SC5314 genomic DNA containing homology to the pNAT plasmid. Primers KL11 and KL12 were used to amplify the *NAT* selection marker from the pNAT plasmid containing homology downstream of the *MIG1* open reading frame. Primers KL42 and KL43 were used to construct the *CmLEU2* single guide RNA. The *CmLEU2* sgRNA, Cas9 PCR, and *MIG1* fused to the *NAT* selection marker were transformed into strain KL742 to generate a *LEU2*-, NAT resistant, heterozygous strain with *MIG1/*mig1*Δ* at the *mig1Δ* locus. *CmLEU2* amplified from SN250 genomic DNA was transformed into the *LEU2*- strain. Transformants were selected on CSM media lacking Leucine.

Strains KL951 and KL952 (*sak1Δ/Δ mig1Δ/Δ*) was constructed using SN152 as the parent strain. Transformation mix contained a *MIG1* sgRNA expression cassette created using primers KL13 and KL14, Cas9, and a *CdHIS1* deletion cassette amplified from pSN52 using primers KL15 and KL16 which contain approximately 80 basepairs of homology upstream or downstream of the *MIG1* coding region. Transformants were selected on CSM media lacking Histidine. The resulting *mig1Δ/Δ* strain was used to create the *sak1Δ/Δ mig1Δ/Δ* strain. Transformation mix contained a *SAK1* sgRNA expression cassette created using primers KL17 and KL18, Cas9, and a *CdARG4* deletion cassette amplified from pSN69 using primers KL19 and KL20 which contain approximately 80 basepairs of homology upstream or downstream of the *SAK1* coding region. Transformants were selected on CSM media lacking Arginine. To create a prototrophic strain, two PCR products created from primers KL21 and KL22 and KL23 and KL24 were used to PCR *CmLEU2* from genomic DNA from strain SN250 and were transformed into the resulting strain to add *CmLEU2* to the *C. albicans leu2Δ* locus. Transformants were selected on CSM media lacking Leucine.

Strains KL953 and KL954 (*snf1Δ/Δ mig1Δ/Δ*) was constructed using SN152 as the parent strain. Transformation mix contained a *MIG1* sgRNA expression cassette created using primers KL13 and KL14, Cas9, and a deletion cassette amplified from pSN52 using primers KL15 and KL16 which contain approximately 80 basepairs of homology upstream or downstream of the *MIG1* coding region. Transformants were selected on CSM media lacking Histidine. The resulting *mig1Δ/Δ* strain was used to create the *snf1Δ/Δ mig1Δ/Δ* strain. Transformation mix contained a *SNF1* sgRNA expression cassette created using primers KL25 and KL26, Cas9, and a *CdARG4* deletion cassette amplified from pSN69 using primers KL27 and KL28 which contain approximately 80 basepairs of homology upstream or downstream of the *SNF1* coding region. Transformants were selected on CSM media lacking Arginine. To create a prototrophic strain, two PCR products created from primers KL21 and KL22 and KL23 and KL24 were used to PCR *CmLEU2* from genomic DNA from strain SN250 and were transformed into the resulting strain to add *CmLEU2* to the *C. albicans leu2Δ* locus. Transformants were selected on CSM media lacking Leucine.

KL955 (*sak1Δ/Δ mig1Δ/Δ mig2Δ/Δ*) was constructed using the *LEU2-* parent strain of KL951. Transformation mix contained a *MIG2* sgRNA expression cassette created using primers KL1 and KL2, Cas9, and a *LEU2* deletion cassette amplified from pSN40 using primers KL3 and KL4 which contain approximately 80 basepairs of homology upstream or downstream of the *MIG2* coding region. Transformants were selected on CSM media lacking Leucine.

KL957 (*snf1Δ/Δ mig1Δ/Δ mig2Δ/Δ*) was constructed using the *LEU2-* parent strain of KL953. Transformation mix contained a *MIG2* sgRNA expression cassette created using primers KL1 and KL2, Cas9, and a *LEU2* deletion cassette amplified from pSN40 using primers KL3 and KL4 which contain approximately 80 basepairs of homology upstream or downstream of the *MIG2* coding region. Transformants were selected on CSM media lacking Leucine.

KL960 and KL961 (*sak1Δ/Δ mig2Δ/Δ*) was constructed using SN152 as the parent strain. Transformation mix contained a *MIG2* sgRNA expression cassette created using primers KL13 and KL14, Cas9, and a deletion cassette amplified from pSN40 using primers KL15 and KL16 which contain approximately 80 basepairs of homology upstream or downstream of the *MIG2* coding region. Transformants were selected on CSM media lacking Leucine. The resulting *mig2Δ/Δ* strain was used to create the *sak1Δ/Δ mig2Δ/Δ* strain. Transformation mix contained a *SAK1* sgRNA expression cassette created using primers KL17 and KL18, Cas9, and a *CdARG4* deletion cassette amplified from pSN69 using primers KL19 and KL20 which contain approximately 80 basepairs of homology upstream or downstream of the *SAK1* coding region. Transformants were selected on CSM media lacking Arginine. To create a prototrophic strain, two PCR products created from primers KL21 and KL29 and KL23 and KL30 were used to PCR *CdHIS1* from genomic DNA from strain SN250 and were transformed into the resulting strain to add *CdHIS1* to the *C. albicans leu2Δ* locus. Transformants were selected on CSM media lacking Histidine.

KL988 (sak1Δ/Δ) was constructed using SN250 as the parent strain. Transformation mix contained a *SAK1* sgRNA expression cassette created using primers KL17 and KL18, Cas9, and a *NAT* deletion cassette amplified from pNAT using primers KL19 and KL20 which contain approximately 80 basepairs of homology upstream or downstream of the *SAK1* coding region. To create a prototrophic strain, one copy of *C. albicans* *ARG4* amplified from SC5431 genomic DNA was integrated at the *arg4Δ* locus. Transformants were selected on CSM media lacking Arginine.

KL992 (sak1Δ/Δ + SAK1) was constructed using the *ARG4-* parent strain of KL988. To validate the *sak1Δ/Δ* strain, primers KL31 and KL32 were used to amplify the *SAK1* allele from SC5314 genomic DNA, containing homology to pSN69. Primers KL32 and KL11 were used to amplify the *cdARG4* deletion cassette from pSN69, containing homology downstream of the *SAK1* coding region. A single guide expression cassette targeting the *NAT* deletion cassette at the *sak1Δ* locus was created using primers KL34 and KL35 [4]. Transformation mix contained the two PCR products, *NAT* sgRNA, and Cas9. Transformants were selected on CSM media lacking Arginine.

Strains KL972 (*sak1Δ/Δ mig1Δ/Δ + SAK1/SAK1*), KL974 (*sak1Δ/Δ mig1Δ/Δ mig2Δ/Δ + SAK1/SAK1*), and KL990 (*sak1Δ/Δ mig2Δ/Δ + SAK1/SAK1*): To validate the *sak1Δ/Δ* strains, primers KL31 and KL36 were used to amplify the *SAK1* allele from SC5314 genomic DNA, containing homology to pNAT. Primers KL33 and KL11 were used to amplify the *NAT* deletion cassette from pNAT, containing homology downstream of the *SAK1* coding region. A single guide expression cassette targeting the *cdARG4* deletion cassette at the *sak1Δ* locus was created using primers KL37 and KL38. Transformation mix contained the two PCR products, *cdARG4* sgRNA, and Cas9. Transformants were selected for drug resistance on YPD media + nourseothricin and replica plated to select colonies unable to grow on CSM lacking Arginine. To create a prototrophic strain, one copy of *CaARG4* amplified from SC5431 genomic DNA was integrated at the *arg4Δ* locus.

Strains KL970 (*snf1Δ/Δ mig1Δ/Δ + SNF1/SNF1*), and KL976 (*snf1Δ/Δ mig1Δ/Δ mig2Δ/Δ + SNF1/SNF1*): To validate the *snf1Δ/Δ* strains, primers KL41 and KL11 were used to amplify the *SNF1* allele from SC5314 genomic DNA, containing homology to pNAT. Primers KL40 and KL39 were used to amplify the *NAT* deletion cassette from pNAT, containing homology downstream of the *SNF1* coding region. A single guide expression cassette targeting the *cdARG4* deletion cassette at the *snf1Δ* locus was created using primers KL37 and KL38. Transformation mix contained the two PCR products, *cdARG4* sgRNA, and Cas9. Transformants were selected for drug resistance on YPD media + nourseothricin and replica plated to select colonies unable to grow on CSM lacking Arginine. To create a prototrophic strain, one copy of *CaARG4* amplified from SC5431 genomic DNA was integrated at the *arg4Δ* locus.

Literature cited:

1. Homann OR, Dea J, Noble SM, Johnson AD (2009) A phenotypic profile of the Candida albicans regulatory network. PLoS Genet 5: e1000783.

2. Vylkova S, Lorenz MC (2015) Modulation of phagosomal pH by Candida albicans promotes hyphal morphogenesis and requires Stp2p, a regulator of amino acid transport. PLoS Pathog 10: e1003995.

3. Huang MY, Woolford CA, Mitchell AP (2018) Rapid Gene Concatenation for Genetic Rescue of Multigene Mutants in Candida albicans. mSphere 3.

4. Huang MY, Mitchell AP (2017) Marker Recycling in Candida albicans through CRISPR-Cas9-Induced Marker Excision. mSphere 2.
