# Supplementary material for: Roles of Candida albicans Mig1 and Mig2 in glucose repression, pathogenicity traits, and SNF1 essentiality
Source: PLoS Genet. 2020 Jan 21;16(1):e1008582. doi: 10.1371/journal.pgen.1008582 (PMC6994163; doi:10.1371/journal.pgen.1008582)
Supplement: S2 Text — The sequence of plasmid ED3-HA is provided. (DOCX) [file pgen.1008582.s007.docx]

>pED3 (HA-Act1term)

AGCGCCCAATACGCAAACCGCCTCTCCCCGCGCGTTGGCCGATTCATTAATGCAGCTGGCACGACAGGTTTCCCGACTG

GAAAGCGGGCAGTGAGCGCAACGCAATTAATGTGAGTTAGCTCACTCATTAGGCACCCCAGGCTTTACACTTTATGCTT

CCGGCTCGTATGTTGTGTGGAATTGTGAGCGGATAACAATTTCACACAGGAAACAGCTATGACCATGATTACGCCAAGC

TATTTAGGTGACACTATAGAATACTCAAGCTATGCATCAAGCTTTACCCATACGATGTTCCTGACTATGCGGGCTATCC

CTATGACGTCCCGGACTATGCAGGATCCTATCCATATGACGTTCCAGATTACGCTTAAGAGTGAAATTCTGGAAATCTG

GAAATCTGGTTTTGTATTCTTGTTATTCTTCTTTTTGTTATTACATATATAACTTGTTACTTTTTTAAAAAAATCTTTG

TTTATTTTATAAATATATAAAACTAAATTTAAGAAAAAGAGAAAAATGTTTTATTTGAGAGATTGAAATTTTACTTGAA

TTTAGCTTAGCTTTTATAAAGTATTATTATGTAAAAAAACAAAACAAATATACATTAAAAAGTTAAGACTATAAAATAG

CCACCCAAGGCATTTCTATATCTTGTTGTTGTTGTTTTCATCTTCTGTATCAGAGGAACTTATTTTATTATTTTCGTCA

CGGGTATTTTCTCTTGTTTGATGATTCATCCCATTCATTCCATCATAAAATGTCGACCATAATCTAGCAACATCGAGCT

CGGATCCACTAGTAACGGCCGCCAGTGTGCTGGAATTCGCCCTTGTTTGATACCATGGCAAGCATACTAGAATTATTAT

ACGAGCTGGGTGTGAAATGTGCCACCGTTTATGCATTTTCGATCGAAAACTTCAAGAGACTGCAATATGAGGTCAAATG

GTTGATGGAATTAGCCAAATCCAAGTTTACGCAAATAAACCAACATGGATTGTTGTGTGAGGAGTATGGGGTTAAAATC

AGAATATTAGGAAATACCAAACTTTTGCCGAAAGATGTGTTAGAGATCCTTGAAAGAACCGAAGAAATAACTAAAAACA

ATAAGCGTGCTGTGTTGAATGTCTGCTTCCCATATACATCAAGAGACGAGATAACCCATCTGATCAAGTCAGTAGTAGA

CGAAGCGACGAAAGGTGGTATTGAAATAACCGAGGAGGCTATCGACAAGCATCTTTATACGAAAGATGTGCCCCCATTG

GATTTACTAGTAAGAACATCAGGAACATTTAGACTTTCAGACTTTCTTTTATGGCAAACGGTGTCGCCAGATTGTGCAG

TTGTGTTTGTCGACAAGTTATGGCCGGCATTCTAGACCATGGGATATGCTCAAGATTTTACTCAACTGGGGGTTTAACA

AATACATGTATGGAAACCCAAATGGATATGGGGTTAGTAATAACTTGGTAGTGAAGAGCCAAATGGAAGAAGAATTAAT

AAACACACCAAATGGAGCTACAGGGCTTGACCGGTACCTGGAGGACGAAGAGACCGAAGTTAGTAGTAACAATTTAGGA

ACTGAAGAGGATACAGTGACATCCGAAGAAGAAGATGTTGAAGATATAGGCAAAAAGTAATAGGACAGTATTTTATTTA

TTCAATAGTTTTTTTTTTTTTTTTGAAACGTACACCTTGTTTATTTCAGGTGTTTCTTTGAAACGTGTCTCAAAGCGAT

AACGGGTACAAAAAAAAATGTATTGAGTCATTGAAATTTTTTTATTTTTTTTTTTTTTTGGTGAAGATTTTTCCCACAC

CAAATATATACCACTTTTCTTCTAACACCACCTACATATTCACTATGGATTTAGTCAATCATTTGCCCGATCGTTTGTT

ATTTGCTGTTCCCAAAAAGGGCAGATTATATGAAAAATGCTGCAGCTTATTGAGCGGTGCCGATATACAGTTTAGAAGA

TCTAATAGATTGGATATTGCCCTTTCCACAAACTTGCCAATTGCATTAATCTTTTTGCCTGCAGCCGATATTCCAGTTT

TCGTTGGAGAAGGTAATTGTGACTTGGGTATAACTGGGTTAGACCAGATCAAAGAAGCAGACCAATTCGACAACATTGA

GGATTTGTTGGATTTGAAGTTTGGCTCATGTAAATTGCAGATCCAAGTTCCTGCAAATGGCGAGTACGAAAAACCCGAA

CAACTCGTTGGAAAGAAGATTGTATCCTCCTTTACAAAGTTGAGTACTGACTATTTCAAGAAATTATCCGACAAACCTA

CGAATATTAGATACGTTGGTGGTTCAGTTGAGGCTTCTTGCGCATTGGGTGTTGCCGACGCCATTGTCGATTTAGTTGA

AAGTGGAGAGACCATGAAAGCTGCTGGATTAAAGGCGATCGAAACCATATTGGAAACTTCCGCTCATTTGATTTCCTCC

AAGAAAACCAAATTTCCAGAAATGGTCAACATAATTGTCCAAAGACTTCAAGGTGTTTTAGCGGCTCAGGAATATGTCT

TGTGTAACTACAATGCTCCAAAATCCATTCAATCAAAGTGCTTAACCATTACTCCAGGTAGAAGGGCAGCCACTGTCTC

CACTTTGGATAAACACAGTGACGACGAAGATGACTGGGTTGCCATTTCTTCCATGGTTAACAGAAAGGAAATTGGTAAT

GTAATGGATGAATTGAAGAAAGCTGGCGCAACAGATATATTGGTGCTCGAGATATCAAATTGTAGAGTTTAATAAAATA

CTATATATACATACTTATAGATTTACTTCAATTTGACTCAATCTGTCTTATACCATCTTTATCTACCACCTTGATGTAC

ACACCTTTAAAATCAATGGGCATTCTCGTTTGTAATTCTTTGACACACATGTCCATTAACTTCAAACCATCTTCAACTG

TCATGTCCTTTCTATAATGTTTATCTAACAAGGATGTTGTGTAAAATGCTGCGTAGCCATGAGCACCATAAGGACGTTC

AACTTGCGTACCTAAATAATCTATCCAATTCAACGACGGCTGATTTGTCTTTACATCGTATCCTCCAATTAAACAGTTC

ACCTGGTACGGTTTTCTAGAAGGGCGAATTCTGCAGATATCCATCACACTGGCGGCCGCTCGAGCATGCATCTAGAGGG

CCCAATTCGCCCTATAGTGAGTCGTATTACAATTCACTGGCCGTCGTTTTACAACGTCGTGACTGGGAAAACCCTGGCG

TTACCCAACTTAATCGCCTTGCAGCACATCCCCCTTTCGCCAGCTGGCGTAATAGCGAAGAGGCCCGCACCGATCGCCC

TTCCCAACAGTTGCGCAGCCTATACGTACGGCAGTTTAAGGTTTACACCTATAAAAGAGAGAGCCGTTATCGTCTGTTT

GTGGATGTACAGAGTGATATTATTGACACGCCGGGGCGACGGATGGTGATCCCCCTGGCCAGTGCACGTCTGCTGTCAG

ATAAAGTCTCCCGTGAACTTTACCCGGTGGTGCATATCGGGGATGAAAGCTGGCGCATGATGACCACCGATATGGCCAG

TGTGCCGGTCTCCGTTATCGGGGAAGAAGTGGCTGATCTCAGCCACCGCGAAAATGACATCAAAAACGCCATTAACCTG

ATGTTCTGGGGAATATAAATGTCAGGCATGAGATTATCAAAAAGGATCTTCACCTAGATCCTTTTCACGTAGAAAGCCA

GTCCGCAGAAACGGTGCTGACCCCGGATGAATGTCAGCTACTGGGCTATCTGGACAAGGGAAAACGCAAGCGCAAAGAG

AAAGCAGGTAGCTTGCAGTGGGCTTACATGGCGATAGCTAGACTGGGCGGTTTTATGGACAGCAAGCGAACCGGAATTG

CCAGCTGGGGCGCCCTCTGGTAAGGTTGGGAAGCCCTGCAAAGTAAACTGGATGGCTTTCTCGCCGCCAAGGATCTGAT

GGCGCAGGGGATCAAGCTCTGATCAAGAGACAGGATGAGGATCGTTTCGCATGATTGAACAAGATGGATTGCACGCAGG

TTCTCCGGCCGCTTGGGTGGAGAGGCTATTCGGCTATGACTGGGCACAACAGACAATCGGCTGCTCTGATGCCGCCGTG

TTCCGGCTGTCAGCGCAGGGGCGCCCGGTTCTTTTTGTCAAGACCGACCTGTCCGGTGCCCTGAATGAACTGCAAGACG

AGGCAGCGCGGCTATCGTGGCTGGCCACGACGGGCGTTCCTTGCGCAGCTGTGCTCGACGTTGTCACTGAAGCGGGAAG

GGACTGGCTGCTATTGGGCGAAGTGCCGGGGCAGGATCTCCTGTCATCTCACCTTGCTCCTGCCGAGAAAGTATCCATC

ATGGCTGATGCAATGCGGCGGCTGCATACGCTTGATCCGGCTACCTGCCCATTCGACCACCAAGCGAAACATCGCATCG

AGCGAGCACGTACTCGGATGGAAGCCGGTCTTGTCGATCAGGATGATCTGGACGAAGAGCATCAGGGGCTCGCGCCAGC

CGAACTGTTCGCCAGGCTCAAGGCGAGCATGCCCGACGGCGAGGATCTCGTCGTGACCCATGGCGATGCCTGCTTGCCG

AATATCATGGTGGAAAATGGCCGCTTTTCTGGATTCATCGACTGTGGCCGGCTGGGTGTGGCGGACCGCTATCAGGACA

TAGCGTTGGCTACCCGTGATATTGCTGAAGAGCTTGGCGGCGAATGGGCTGACCGCTTCCTCGTGCTTTACGGTATCGC

CGCTCCCGATTCGCAGCGCATCGCCTTCTATCGCCTTCTTGACGAGTTCTTCTGAATTATTAACGCTTACAATTTCCTG

ATGCGGTATTTTCTCCTTACGCATCTGTGCGGTATTTCACACCGCATACAGGTGGCACTTTTCGGGGAAATGTGCGCGG

AACCCCTATTTGTTTATTTTTCTAAATACATTCAAATATGTATCCGCTCATGAGACAATAACCCTGATAAATGCTTCAA

TAATAGCACGTGAGGAGGGCCACCATGGCCAAGTTGACCAGTGCCGTTCCGGTGCTCACCGCGCGCGACGTCGCCGGAG

CGGTCGAGTTCTGGACCGACCGGCTCGGGTTCTCCCGGGACTTCGTGGAGGACGACTTCGCCGGTGTGGTCCGGGACGA

CGTGACCCTGTTCATCAGCGCGGTCCAGGACCAGGTGGTGCCGGACAACACCCTGGCCTGGGTGTGGGTGCGCGGCCTG

GACGAGCTGTACGCCGAGTGGTCGGAGGTCGTGTCCACGAACTTCCGGGACGCCTCCGGGCCGGCCATGACCGAGATCG

GCGAGCAGCCGTGGGGGCGGGAGTTCGCCCTGCGCGACCCGGCCGGCAACTGCGTGCACTTCGTGGCCGAGGAGCAGGA

CTGACACGTGCTAAAACTTCATTTTTAATTTAAAAGGATCTAGGTGAAGATCCTTTTTGATAATCTCATGACCAAAATC

CCTTAACGTGAGTTTTCGTTCCACTGAGCGTCAGACCCCGTAGAAAAGATCAAAGGATCTTCTTGAGATCCTTTTTTTC

TGCGCGTAATCTGCTGCTTGCAAACAAAAAAACCACCGCTACCAGCGGTGGTTTGTTTGCCGGATCAAGAGCTACCAAC

TCTTTTTCCGAAGGTAACTGGCTTCAGCAGAGCGCAGATACCAAATACTGTCCTTCTAGTGTAGCCGTAGTTAGGCCAC

CACTTCAAGAACTCTGTAGCACCGCCTACATACCTCGCTCTGCTAATCCTGTTACCAGTGGCTGCTGCCAGTGGCGATA

AGTCGTGTCTTACCGGGTTGGACTCAAGACGATAGTTACCGGATAAGGCGCAGCGGTCGGGCTGAACGGGGGGTTCGTG

CACACAGCCCAGCTTGGAGCGAACGACCTACACCGAACTGAGATACCTACAGCGTGAGCTATGAGAAAGCGCCACGCTT

CCCGAAGGGAGAAAGGCGGACAGGTATCCGGTAAGCGGCAGGGTCGGAACAGGAGAGCGCACGAGGGAGCTTCCAGGGG

GAAACGCCTGGTATCTTTATAGTCCTGTCGGGTTTCGCCACCTCTGACTTGAGCGTCGATTTTTGTGATGCTCGTCAGG

GGGGCGGAGCCTATGGAAAAACGCCAGCAACGCGGCCTTTTTACGGTTCCTGGGCTTTTGCTGGCCTTTTGCTCACATG

TTCTTTCCTGCGTTATCCCCTGATTCTGTGGATAACCGTATTACCGCCTTTGAGTGAGCTGATACCGCTCGCCGCAGCC

GAACGACCGAGCGCAGCGAGTCAGTGAGCGAGGAAGCGGAAG
