# Supplementary material for: Roles of Candida albicans Mig1 and Mig2 in glucose repression, pathogenicity traits, and SNF1 essentiality
Source: PLoS Genet. 2020 Jan 21;16(1):e1008582. doi: 10.1371/journal.pgen.1008582 (PMC6994163; doi:10.1371/journal.pgen.1008582)
Supplement: S1 Fig — Venn Diagram includes upregulated genes from mig1Δ/Δ mig2Δ/Δ vs wild-type YPD, wild-type YPG vs wild-type YPD, wild-type engulfed by macrophages vs non-engulfed [28], pH4 vs YPD [29], and biofilm cells vs yeast cells [30]. A list of the 116 shared genes can be found in tab S1G of S1 Table. The gene set is enriched for the GO terms “monocarboxylic acid catabolic process,” “fatty acid catabolic process,” and related terms (tab S2D of S2 Table). (PDF) [file pgen.1008582.s001.pdf]

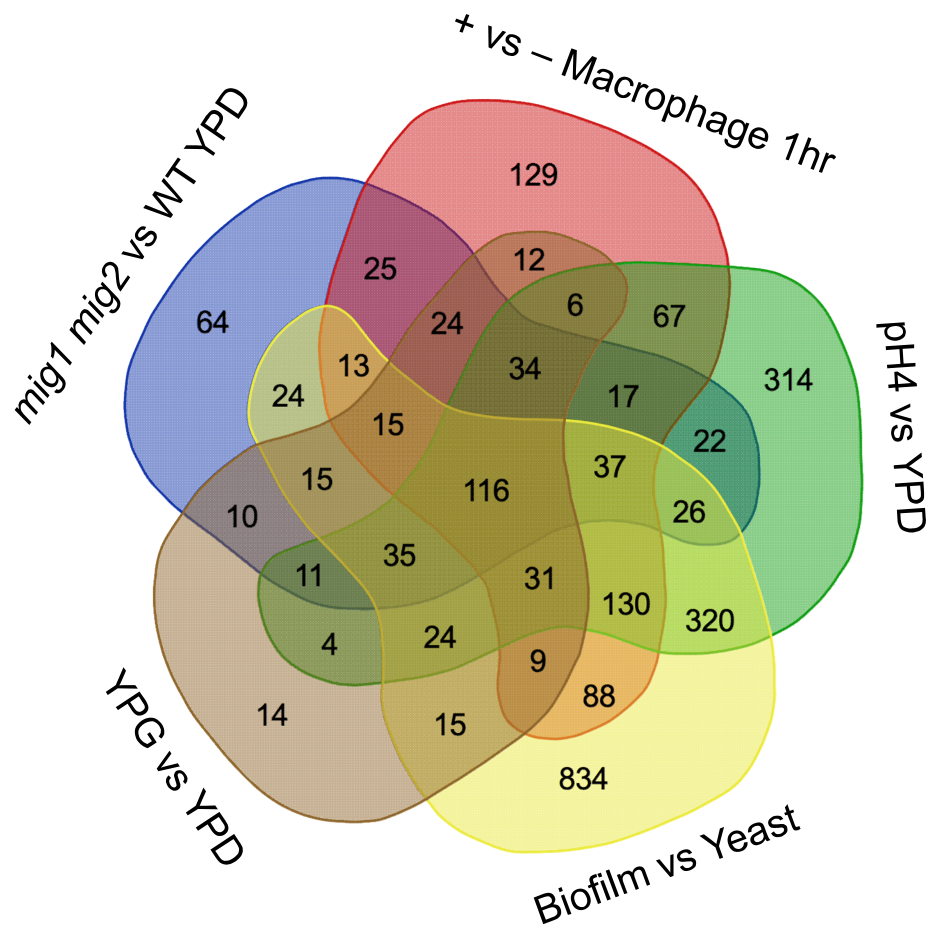

Figure S1. Intersections of YPG vs YPD responsive genes and related datasets. Venn Diagram includes upregulated genes from *mig1Δ/Δ mig2Δ/Δ* vs wild-type YPD, wild-type YPG vs wild-type YPD, wild-type engulfed by macrophages vs non-engulfed [28], pH4 vs YPD [29], and biofilm cells vs yeast cells [30]. A list of the 116 shared genes can be found in tab S1G of S1 Table. The gene set is enriched for the GO terms "monocarboxylic acid catabolic process," "fatty acid catabolic process," and related terms (tab S2D of S2 Table).
