# Supplementary material for: Roles of Candida albicans Mig1 and Mig2 in glucose repression, pathogenicity traits, and SNF1 essentiality
Source: PLoS Genet. 2020 Jan 21;16(1):e1008582. doi: 10.1371/journal.pgen.1008582 (PMC6994163; doi:10.1371/journal.pgen.1008582)
Supplement: S2 Fig — Protein sequences were aligned using Clustal Omega and visualized using Jalview Clustal color scheme. (PDF) [file pgen.1008582.s002.pdf]

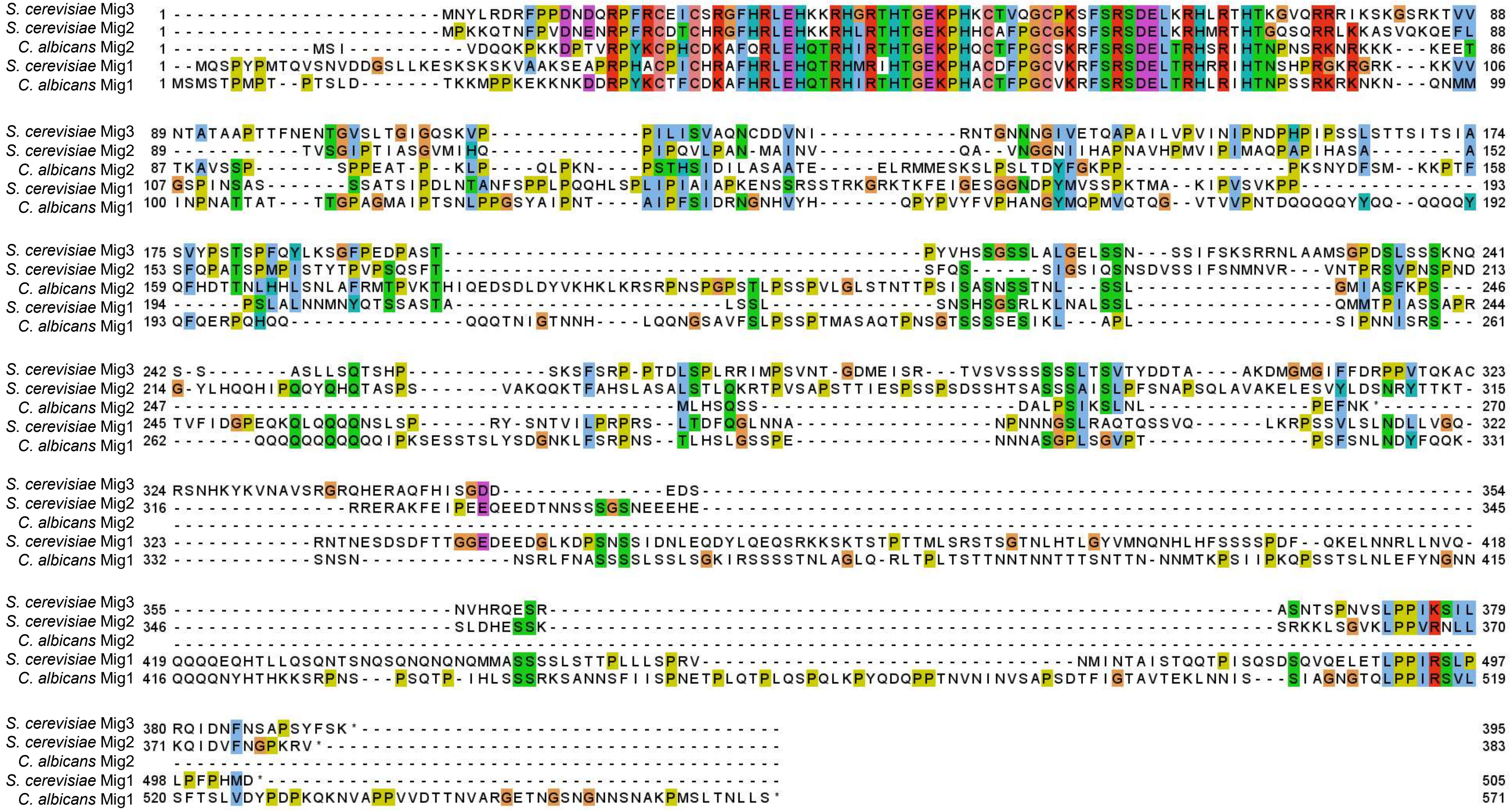

Figure S2. Multiple Sequence Alignment of Mig proteins from *Saccharomyces cerevisiae* and *Candida albicans*.
Protein sequences were aligned using Clustal Omega and visualized using Jalview Clustal color scheme.
