# Supplementary material for: Roles of Candida albicans Mig1 and Mig2 in glucose repression, pathogenicity traits, and SNF1 essentiality
Source: PLoS Genet. 2020 Jan 21;16(1):e1008582. doi: 10.1371/journal.pgen.1008582 (PMC6994163; doi:10.1371/journal.pgen.1008582)
Supplement: Attachment — Submitted filename: Response to Reviews.pdf [file pgen.1008582.s016.pdf]

Response to Reviews

We thank the reviewers for their thorough and thoughtful evaluation of our manuscript. We have made substantial edits to our manuscript to address the reviewers' comments and to make overall improvements to our study. At the editorial level, we have focused the presentation to emphasize more clearly the unique aspects of Mig1/2 function in *C. albicans.* At the experimental level, we have included new results that show that Mig1 protein accumulation is regulated post-transcriptionally in response to carbon source (Figs 3 and S3), a feature more closely aligned with regulation of ScMig2 than that of ScMig1.

Many thanks for considering this revised manuscript. The detailed responses to reviewer comments may be found below.

Reviewer #1: The manuscript by Mitchell et al describes the role of Mig1 and Mig2, two repressors that repress genes involved in carbon source utilization and catabolite repression. The authors show that Sak1 is required for growth in the absence of glucose, and that Mig1 and Mig2 are epistatic to Sak1, in a partially redundant way. The paper begins with a transcriptomics analysis of cells grown in the presence of either glucose of glycerol and includes mig1, mig2, or mig1mig2 mutants, continues to nicely phenotype double and triple mutants along with complemented derivatives to support their genetic model, then characterizes the role of Mig1 and Mig2 in several important phenotypes. The manuscript is well-written and tells a nice story, and provides a satisfying explanation for the essentiality of Snf1 in C. albicans.

Thank you so much for this positive appraisal!

I have a few comments that might strengthen the manuscript.

1) The authors points on the relatedness between their data set and other datasets could be more useful with more detail on whether the same genes (a subset) drove the similarity to the differentially expressed genes in other datasets. For example, were similar subsets of genes among those that drove similarity to macrophage experiments and M199-YPD comparisons? Did the Mig1 and Mig2 and Mig1/2 regulons coincide with any of these experiments? The comparison to other datasets might be useful later on in the paper once the Mig regulons have been defined. As written, this part of the paper is rather speculative.

Thank you for this suggestion. We have included a Venn diagram of the overlap among the major datasets discussed (S2 Fig). You can see that there are 116 genes at the intersection of the major datasets. The gene set is enriched for the GO terms "monocarboxylic acid catabolic process," "fatty acid catabolic process," and related terms (see new tab S2D in S2 Table).

These terms reflect alternative carbon source functions, which would reasonably be expected to be under glucose repression. The genes are listed in a new tab S1G in S1 Table.

2) On p. 14, the following text is found: "Growth properties of representative snf1Δ/Δ derivatives of mig1Δ/Δ and mig1Δ/Δ mig2Δ/Δ strains suggested that loss of Mig1 does not bypass the need for Snf1 entirely. For example, snf1Δ/Δ mig1Δ/Δ colonies exhibited hyperfilamentation and a peculiar yellow color, whereas snf1Δ/Δ mig1Δ/Δ mig2Δ/Δ colonies did not (Fig S1)."
The authors might consider whether the yellow color is riboflavin. Several studies have shown that metabolic perturbation and alterations in cAMP signaling can change riboflavin production. Here is one paper that references riboflavin production but it is not the first (PMID:21992526). Shining a UV light on the colony may reveal characteristic fluorescence and a simple extraction can be analyzed for the reduced and oxidized spectra characteristic of this molecule.

We really liked this idea! However, I regret that our attempts to detect riboflavin in response to this suggestion were negative. Specifically, the colonies did not appear more fluorescent than the wild-type when a UV was shined on colonies grown on YPD plates, and upon extraction, the spectrum of the snf1 mig1 mutant was not significantly different. Our hope is that our work invites renewed interest in Snf1 essentiality, since its suppression by *mig1/2* mutations offers a road to mechanistic dissection, and then we or our colleagues in the field will build upon this yellow color observation further.

3) Fig. 4 should be revised to have genes in all capitals in the right-hand column and the text resolution is of a lesser quality than for other figures.

We apologize for this oversight and have fixed both issues.

4) Italics needed in at least one figure legend and references need to be joined within a single bracket in the introduction.

We apologize for this oversight and have fixed both issues.

5) Could the authors expand on the statement that "peroxisome biogenesis [is a] functions that align well with alternative carbon source utilization".

We agree that a clearer rationale will be helpful for our readers. We have added the statement (line 496) "In yeast, peroxisomes function as sites for hydrogen peroxide reduction and fatty acid metabolism [54], and the abundance of these specialized organelles increases in response to non-glucose media such as oleic acid [55]."

6) Do the transcriptomics data still need to be deposited?

The RNA-sequencing data has been deposited at the SRA database and can be accessed using accession number for RNA-Seq data: PRJNA573104.

Reviewer #2: Regulation of sugar usage is very important for cells, and investigating the mechanisms by which organisms control which sugars are metabolised is a significant scientific endeavor. In the manuscript the Mitchell and Filler labs investigate the roles of Mig1 and Mig2 in glucose repression in the fungal pathogen C. albicans. The basic strategy is to make single and double mutants, and investigate the phenotypes in terms of sugar usage (glucose vs alternative sources) and, because the organism is a pathogen, the consequences for pathogenicity and traits associated with this lifestyle.

In the well-investigated model yeast S. cerevisiae, glucose repression has been connected to the Snf1 kinase and its regulation of the key transcription factor Mig1 – Mig1 serves as a repressor of alternative carbon sources. High glucose, inactive Snf1, active Mig1, and repression of the use of alternative sugars; low glucose, active Snf1, inactive repressor Mig1, and alternate sugars can be used. There are lots of nuances and other players in the circuitry, but the Snf1/Mig1 interaction is central to the control process. However, S. cerevisiae is somewhat of an outlier in aspects of carbon utilization relative to other fungi, in particular its propensity to ferment glucose even in the presence of oxygen (the Crabtree/Warburg effect), so it is reasonable to ask if the glucose circuitry is also controlled by Snf1/Mig1 in a non-Crabtree yeast like C. albicans.

Basically, the results of this study say yes – the Snf/Mig regulatory circuit controls alternative carbon use in C. albicans.

Thank you for this clear distillation of our manuscript!

First, the authors do an RNAseq analysis of glucose vs glycerol grown cells and identify gene sets that are up-regulated (~ 400) and down-regulated (~ 100) in response to the growth in glycerol. These genes are compared to the expected genes from studies in S. cerevisiae, and to data sets for other comparisons in C. albicans, and are found to represent a sensible data set. Thus C. albicans appears to have a "standard" glucose repression response.

Next, they examine whether Mig1 and Mig2, two paralogs of the Mig1/2 repressors in S. cerevisiae, are important for glucose repression. The

phylogenetic relationships of Mig1/2 in S. cerevisiae and Mig1/2 in C. albicans needs to be better explained. Essentially, as C. albicans is a pre WGD yeast, and S. cerevisiae is a post WGD yeast, there are several possible ways you could get a Mig1 and a Mig2 in both species. It is worth looking to see if a prediction can be made about whether the pre WGD yeast had a Mig1 and a Mig2 that became Mig1A and Mig1B; Mig2A and Mig2B after the duplication and resolved back to Mig1A and Mig2B, or did it resolve to Mig1A, Mig1B in yeast and lose the whole Mig2 lineage from before the WGD? Lots of possible arrangements, worth seeing if something could be said, especially given that the Mig1 and Mig2 in C. albicans seem to be functionally closer to each other than the Mig1 and Mig2 of S. cerevisiae.

Thank you for this suggestion. We conducted a CLUSTAL alignment among the *C. albicans* and *S. cerevisiae* Mig proteins (S2 Fig). The outcome is that CaMig2 is more closely related to ScMig2/3 than to ScMig1. CaMig1 is more closely related to ScMig1 than to ScMig2/3. Hence this analysis suggests that *C. albicans* does not have two Mig1's or two Mig2's.

However, our new experimental results (lines 281-300) provide an interesting twist to the story in this context. Specifically, we find that CaMig1 is regulated post-transcriptionally at the level of protein accumulation, a mode of regulation that is more akin to that of ScMig2 (protein degradation) than ScMig1 (nuclear vs cytoplasmic localization). We were unable to detect epitope-tagged CaMig2, so we cannot say whether the regulatory modes may be reversed relative to *S. cerevisiae,* but this new result suggests that, in terms of regulatory mechanism, CaMig1 is more closely related to ScMig2 than to ScMig1.

To investigate the functions of the two candidate repressors, they make the single and double mutants, complement the genes by dropping versions in at the standard RPS1 locus, and, in the cases where position effects prevent full complementation, they replace to WT gene at the WT locus. This provides confidence the phenotypic assessments to follow are really the result of the loss of Mig1 or Mig2 function.

The first set of phenotypes is to see which genes are de-repressed in the absence of either or both the repressors. The authors identify genes that are repressed by both (need to be mig1-mig2- to see de-repression), repressed specifically by Mig1 (de-repressed in the mig1- or the mig1-mig2- strains), and repressed specifically by Mig2. The suggest that the specifically repressed genes can be functionally sorted – Mig2 for glyoxylate and beta oxidation, Mig1 for transporters, Mig1 and Mig2 for everything else, and in fact the majority of the regulated genes. Finally, they argue the Mig1/2 regulated genes are the same genes that show glucose repression, and that glucose repression of these genes doesn't work well in the double mutant. These data sets do not correlate

perfectly of course, and the failure of glucose repressed genes to respond in the mig1/2 double mutant is not absolute (still some apparent repression of some genes), but the patterns seen strongly support the central premise here.

Thank you for this distillation and summary. There are always exceptions to regulatory relationships, and we are pleased that our main messages were conveyed.

Next the authors address the relationship of Snf1 and the Mig1/2 elements. Loss of Snf1 is lethal to C. albicans cells, this is somewhat surprising and has greatly limited analysis of the element. Based on the hypothesis that over-suppression of the Mig gene targets could be the cause of the lethality, the authors attempted to rescue the snf1 nulls be deleting the repressors, and were able to get viable snf1 nulls in the mig1- and mig1-mig2- backgrounds. Thus, overactivity of Mig1, presumably suppression of some essential function(s), appears to be the cause of the Snf1 null's inviability. They follow this up by assessing the impact of the mig mutants on the regulatory kinase upstream of Snf1, and show that growth defects associated with loss of this kinase are abrogated by the repressor deletions, but find that the mig2 repressor is more important, in contrast to mig1 which is the cause of the snf1 inviability.

The authors then use nanostring probes for a set of genes defined as mig1, mig2 or mig1/mig2 responsive, and probe the consequences of various perturbations. Implementing the Sak1 response needs both mig1 and mig2, but there are genes that seem to specifically require one or the other. Snf1 also seems to go through mig1/2, with some specific differences from the Sak1 data set.

Other phenotypes are probed – response to wall stress, ability to make hyphae, to interact with immune and endothelial cells. Mig1/2 function is needed, either gene alone does a pretty good job, so the proteins appear close to redundant for these functions. The data in general are convincing and sensibly interpreted (with the caveats noted below), and the results suggest logical explanations for otherwise somewhat surprising results such as the difference in snf1 and sak1 phenotypes and the essentiality of snf1.

My major concern with this work is the context in which it is embedded. At one level, the sak1, snf1, mig1 circuit controls glucose repression in C. albicans essentially as it does in S. cerevisiae, so nothing fundamental has been changed in or added to our understanding of the process. To justify the work to the audience of PLoS Genetics the

authors want to note the way C. albicans has structured the circuit relative to S. cerevisiae – to emphasize the differences. But here they seem to somewhat lose their way. The fact that in C. albicans the paralogs Mig1 and Mig2 act similarly, but have a selective bias in targets is stated as surprising, when it is exactly what should happen with paralogs in this situation; they diverge in function, but modestly. The suggestion that the trajectory of Mig function "evolution" is that it has expanded in C. albicans relative to S. cerevisiae seems less likely than that the arrangement in the post WGD yeast S. cerevisiae has compacted relative to the pre WGD yeast C. albicans. If the authors are going down this route they should be providing genomic evidence from phylogenetically intermediary species, and showing the real relationships among the Mig orthologs, as previously noted.

Thank you for this clear criticism. As we read the critiques we began to realize that our presentation included a lot of distractions, including some sloppy language regarding evolution, that ultimately obscured the emphasis that we hoped to achieve. We have rewritten the entire manuscript from Title through Discussion to try to zero in on what we think is interesting and significant. Please read the revised abstract to get a sense of our view of the context.

Minor points

The authors have left PMID identifiers instead of actual references in a few places.

We have gone through the revision carefully to fix those citations. Thanks for catching them.

Reviewer #3: This work addresses the SNF1/ AMP-activated protein kinase signaling system in C. albicans. RNA seq was used to probe the relationship between the transcriptional regulators of carbon source utilization, Mig1 and Mig2, in Candida albicans, and their upstream regulators, the kinases Snf1 and Sak1. Deletion mutants in the genes encoding these regulators were created singly and in combination with each other, and the effects of the mutations on the transcriptional profile of mutants was examined. Nanostring analysis of 28 genes was used to further examine the effects of these mutations on gene expression. Growth, filamentation and biofilm phenotypes of the mutants were analyzed. Lastly, the behavior of mig1 mig2 double mutants during the interaction with model host cells, macrophages and human umbilical vein endothelial cells, was tested.

The authors first established, by RNA seq, the genes differentially expressed in wild type cells growing in the preferred carbon source glucose, or an alternative carbon source, glycerol, in complex rich

medium; they found an expected assortment of genes, the majority of which were upregulated. They next obtained RNA seq datasets for deletion mutants in MIG1, MIG2 and MIG1MIG2 grown in glucose, the carbon source in which the activity of these regulators is predicted to be most important, and again found a majority of genes with increased expression among the total of genes with altered expression levels. Presence of a binding motif for Mig1 (previously established by others) was examined for promoters of genes whose expression was altered in these mutants, and was found to occur at significant frequency in genes whose expression was increased, but not those whose expression was decreased in mig1, mig2 and mig1 mig2 mutants. (No further analysis was attempted of this observation, though an important consideration is whether these regulators control the expression of other transcriptional activators that are responsible for loss of expression of this set of genes.)

Thank you for pointing out the potential role of activators in the gene expression response to *mig1* and *mig2* defects. We have started to characterize several candidate carbon gene activators that are under Mig1/2 control but our results are too preliminary at this time for inclusion. We have considered more generally the roles of Mig1/2-responsive activators in phenotypic outputs in the original manuscript. Specifically, we noted that filamentation regulators Brg1 and Ume6 are under Mig1/2 control (lines 539-542), and that this may account for the filamentation regulation by Mig1/2 that we observed.

The investigators then turned to the specific roles played by Mig1 and Mig2. They found a set of genes whose expression levels were increased only when MIG1 was deleted. Among them were plasma membrane hexose transporters, HGT genes, of which 8 were expressed at increased levels. Genes whose expression increased only when MIG2 was deleted included genes required for lipid catabolism; hence these regulators have distinct specificities in the processes they target.

Most genes whose expression was increased in the mig1 mig2 mutant were unchanged in either of the single mutants; the authors concluded that Mig1 and Mig2 functions largely overlap.

Constructing double mutants in SNF1 with MIG1, MIG2 or MIG1 MIG2, the authors were able to recover snf1 mutants only in a background in which MIG1 was deleted. They then examined growth phenotypes of sak1 mutants and found that their growth defects were suppressed in mutants also lacking MIG2, and further suppressed in when MIG1 was additionally, but not solely, lacking.

The authors then used a panel of 28 nanostring probes to investigate the effects of mutating these regulators singly and in combination. They

observed very small numbers of differentially regulated genes among that set. The mutants were then subjected to cell wall stress using caspofungin, to which mutants lacking Mig1 were hypersensitive. Filamentation and biofilm phenotypes of mig1mig2 double mutants were shown, and the same mutants were defective in damaging macrophages and endothelial cells.

The role of the orthologous genes and their products, in particular SNF1, as an ortholog of the mammalian AMP kinase, has been studied extensively in S. cerevisiae, and in other work in C. albicans. The study is a useful compilation of datasets and shows unique results specific to C. albicans, and as such makes a significant contribution to the field of Candida albicans physiology. Superficiality in experimental design and of the analysis, to some extent limits the potential of this manuscript to move our understanding forward.

The reviewer correctly notes that Snf1 has been studied a fair bit in *C. albicans*. In addition, Mig1 of *C. albicans* has been studied as well, as we noted in our introduction. However, additional regulators of glucose repressible genes remained to be discovered, and Mig2 has not been considered in the published literature to our knowledge. You can see in CGD that Mig2-associated phenotypes have all come out of large-scale mutant screens, and none are directly tied to carbon regulation. Recent authoritative reviews on fungal pathogen carbon regulation by Van Dijck and colleagues (Front Microbiol. 2019; 10: 99.) and by Goldman and colleagues (Mol Microbiol. 2018 Feb;107(3):277-297.) do not mention Mig2 even once. We found that Mig1 and Mig2 together regulate the bulk of glucose-repressible genes in *C. albicans*. On this basis we submit that elucidation of Mig1/2 function fills a significant knowledge gap.

In addition, there has been no insight into the basis for Snf1 essentiality since the initial discovery by Kwon-Chung and colleagues almost 20 years ago. Our work now resolves the two hypotheses put forward by Kwon-Chung. We submit that elucidation of this novel Snf1-Mig1 connection fills a significant knowledge gap.

Major issues:

The analysis is somewhat superficial at several points. E.g.:
1. Is it really an “overlap of the functions” of Mig1 and Mig2 that leads to increased expression of a majority of altered genes in the mig1mig2 double mutant, but not in either single mutant? Certainly redundant activities of these transcriptional regulators could explain this observation. Alternatively, a requirement for cooperative binding at the promoters of these genes, for mutual recruitment, for nucleosome placement etc. (“combinatorial cis-regulation”) (PMID: 19029883) is possible; the authors do not consider any alternative possibilities despite the burgeoning field investigating these regulatory mechanisms,

in which Mig1 is a model. Instead they simply state, for genes whose increased expression in the absence of one of the regulators, is further increased when the other is also absent, “We infer for these genes that Mig1 or Mig2 is the major repressor, but that the other Mig1/2 protein can repress weakly in the absence of the first. Therefore, while Mig 1 nd Mig2 each have some specific target genes, they also have highly redundant roles in gene regulation.”

Perhaps we were unclear about the intended meaning of “redundant functions.” We do not mean to draw any conclusions about a detailed biochemical mechanism of repression. The reason is that we have not done any experiments to address biochemical mechanism. We have examined transcript accumulation changes and cellular phenotypic changes that result from loss of Mig1, Mig2, or both. Our conclusion about redundant function really summarizes our finding that each Mig protein makes a contribution to most of the phenotypes we tested, or in other words that the double mutant has a more severe phenotype than either single mutant. Our intended meaning is in line with the literature; please see Jung et al. (Sci Rep. 2018 May 25;8(1):8132. doi: 10.1038/s41598-018-26405-5) for one example of the many papers that use the term as we have.

This a priori assumption of redundant functions of these transcriptional regulators, in light of today’s large literature on this subject - (E.g.: “Understanding these rules, which are often referred to as ‘cis-regulatory grammar’, is a major challenge in modern biology,” PMID: 30570483 or “discuss how different strategies, including extensive cooperative regulation (both direct and indirect), progressive priming of regulatory elements, and the integration of activities from multiple enhancers, confer specificity and robustness to transcriptional regulation during development” PMID: 22868264) - does not seem to be a sufficient consideration of the regulatory mechanism involved.

Thank you for this suggestion. We have modified our summary figure (revision Fig 8) to present a more mechanistic perspective. However, for our overall presentation, we tried to stay fairly close to our data because, as mentioned above, our findings are significant in the context of biology and network relationships.

If the “overlap of functions” idea is correct, why is the expression of 14% of genes, whose expression is higher, in mig1 or mig2 single mutants or in both than in wild type, not higher in the mig1 mig2 mutant (38/268) (Fig. 2A)? Among the genes expressed at higher levels in the mutants than the wild type, this fraction amounts to fully one third (72/216)?

It is true that we neglected to discuss some of the Mig1/2-responsive genes. In response to this criticism we have added the following paragraph to the Discussion (lines 508-514):

“Some genes respond only in the *mig1Δ/Δ* or *mig2Δ/Δ* single mutants, and not in the *mig1Δ/Δ mig2Δ/Δ* double mutant. Most of these genes are down-regulated in the *mig* mutants. Because Mig1 and Mig2 seem to function primarily as repressors, we infer that down-regulated genes respond indirectly to Mig1/2 through compensatory responses to altered metabolism or other biological processes. About 10% of up-regulated genes respond only in one single mutant, and we suggest that they reflect compensatory responses as well.”

Similarly, the sentences starting with “The majority of genes that are up-regulated in the mig1D/D mig2D/D double mutant are not up-regulated …” and ending with “and we refer to them as Mig1/2 shared genes” seem very unclear at best.

Apologies; we have changed the sentence for improved clarity (lines 486-490):

“The majority of genes that are up-regulated in a *mig1Δ/Δ mig2Δ/Δ* double mutant strain show little expression change in either a *mig1Δ/Δ* or *mig2Δ/Δ* single mutant strain. These 258 genes (Fig 2A) represent targets that can be fully repressed by either Mig1 or Mig2. The large number of such genes argues that Mig1 and Mig2 most often function interchangeably.”

2. As 8 HGT genes (though I count 10 in Fig. 2C?) are expressed at elevated levels in mig1 mutants growing in glucose, Mig1 seems to act as a repressor of these genes in glucose. Is it worth considering which hexoses may the products of these genes transport as apparently it is unlikely to be glucose? Or if it is glucose, why does Mig1 normally repress them while cells are growing in glucose?

Thank you for pointing out the confusing issue with the figure and also for raising these interesting questions. With regard to the figure (Fig 2C), we have modified it to show only the putative hexose transporter genes that respond as Mig1-selective. Apologies.

With regard to Hgt functions, it is not really understood what the substrates may be. Two Hgt’s seem to be glucose sensors rather than transporters, Hgt4 and Hgt12, and they may be glucose-repressed in order to limit signal strength. *HGT7* is induced by galactose and has been suggested to be a galactose transporter. But most Hgts do not have experimentally demonstrated substrate specificity, and in fact that knowledge gap even exists to this day for many of the 22 *S. cerevisiae* glucose transporter-like proteins (Hxt’s).

I think it is a very interesting question regarding why glucose should repress glucose transporters. Some Hgt’s are alternative carbon source transporters so their repression by glucose makes some physiological sense. But what about the true transporters of glucose? Let me try an idea out on you: the Hgts are all MFS-type transporters, so they can only import glucose down a concentration gradient. With that in mind, it does the cell no good to transport glucose at a higher rate than the catabolic rate, because they cannot store cytoplasmic glucose

at a concentration above the external concentration. Of course, they can store glucose-6-phosphate (and derivatives like glucose-1-phosphate or glycogen), but the conversion to G6P consumes an ATP. Hence the cell may benefit more from using ATP for anabolic reactions rather than investing in acquiring excess glucose. Now this is total speculation, and a big stretch from our data, so you can understand why it would be a big digression to include this speculation in the manuscript.

3. Using a panel of only 28 nanostring probes seems insufficient to actually move the understanding of these regulators forward. In fact, only 24 are used, because 4 probes examine the expression of the genes under study, i.e. MIG1, MIG2, SNF1, SAK1. How were these 24 genes chosen? If one were to decide that only 24 genes are sufficient, would one not choose only effectors of the processes under study, e.g. enzymes, while avoiding regulators like the transcription factors TYE7 or ACE2? Having such a small sample of genes in the set, it is inaccurate to write "Nanostring analysis indicated that there may be fairly few Mig1-selective target genes expressed in a snf1D/D mig1D/D mutant."

Apologies for omitting the rationale for Nanostring probes. We added it (lines 352-355):

"To investigate how the phenotypic relationships among these regulators relate at the transcriptional level, we used 28 representative probes for Nanostring analysis from different metabolic pathways (i.e., glycolysis, gluconeogenesis, glyoxylate cycle, fatty-acid catabolism) and selected Mig1/2 target genes from our genome-wide analysis."

With regard to the "Nanostring…may" sentence, we deleted it. I agree that it was quite speculative.

Is measurement of 24 genes adequate to move the field forward? I argue that it is. First, I note that many epistasis tests have been carried out with only one or two reporter genes, and many more have been carried out with cellular or organismal phenotypes that reflect an uncertain number of genes. I suspect that the reviewer does not seek to contest all conclusions drawn from those experiments, which go back to Jacob & Monod and Hawthorne & Douglas and reach forward to the present day. Second, I note that no one has carried out epistasis tests with a *C. albicans snf1Δ/Δ* mutant previously because it is inviable. Our study is the first to report it, because our analysis pointed us toward an explanation for *snf1Δ/Δ* mutant inviability. Hence I argue that we have moved the field forward by starting to unpack this long-standing mystery.

4. How can a statement that "Mutations in MIG1 and MIG2 are epistatic to mutations in SAK1 and SNF1" (Fig. 4 legend) be made when no mutants in MIG1 or MIG2 alone were tested? The only mutants tested in this figure are double and triple mig1 and mig2 mutants with sak1 or snf1 mutations. While for snf1, single mutations in this gene lead to inviability, this

is not the case for sak1. With this experimental design, how can the authors claim that their observed results are not due e.g. to synthetic effects between the mutations? - From S. cerevisiae, it is known that Mig1 and Mig2 act downstream of Sak1 and Snf1 so the statement about epistasis seems very logical to the reader; but if the authors aim to be showing an original result and drawing a legitimate conclusion from their own finding, the experimental design must be such that this conclusion can be drawn. To remind the authors of "epistasis," "For any pair of genes X and Y, we evaluated the level of epistasis by comparing the fitness WXY of the double mutant with the product of the fitness values WX and WY of the corresponding single mutants," (PMID: 15592468)

Apologies, and thanks for raising this point of confusion. I believe that our genotype notations may have been unclear. We have revised the figure and figure legend both in an effort to clarify. Specifically, the "+SAK1" reconstituted strains provide expression data for *mig1, mig2,* and *mig1 mig2* mutant strains. We prefer this approach to simply using the original *mig* mutants because reconstitution confirms that we did not pick up a suppressor mutation that alters gene expression responses in the course of strain manipulation.

5. Issues not considered experimentally e.g. other non-glucose carbon sources, either fermentable (e.g., fructose, galactose, maltose) or nonfermentable (e.g., oleic acid, since Mig2 seemed to target lipid catabolism genes) should at least have been considered in the discussion?

The issue of growth on alternative carbon sources is quite interesting. However, in an effort to focus our manuscript on the most important points that come from our data, we prefer not to include a more speculative paragraph.

6. Would it not have contributed to the analysis to use a loss-of-function strain in SNF1 other than the homozygous deletion that was known to be inviable? (e.g. the heterozygote, a strain expressing a destabilized transcript as in PMID:31118301, etc.)

The use of a *snf1* deletion mutant provides the most compelling evidence that Mig1 activity is responsible for lethality, and that is why we used this allele. It will most definitely be interesting to examine interactions with additional types of *snf1* alleles in the future. For example, we may find Snf1 alterations that mimic the *mig1Δ/Δ* or *mig2Δ/Δ* phenotypes. To make a sense out of such a study, it really needs to be a stand-alone story in its own right.

6. In S. cerevisiae, participation of the heterotrimeric SNF1 holoenzyme in multiple signaling pathways is long established (PMID: 17981722); the Snf1 catalytic subunit complexes not only with a gamma subunity Snf4 but with one of 3 possible beta subunits (Gal83, Sip1 or Sip2) which regulate its subcellular localization. Its regulation involves not just

an upstream activating kinase like Sak1 (or Elm3 or Tok1), but also a specific phosphatase (regulatory subunit Reg1 and the catalytic subunit Glc7) and autoinhibition modulted by the gamma subunit. Among multiple regulatory mechanims including control of transcriptional regulators beyong Mig proteins, Snf1 controls the activity of metabolic enzymes by phosphorylation, such as Pfk27, Gpd2, and Acc1 (PMID: 25512067). SNF1 also is activated in response to stressors like alkaline stress and sodium stress, with differential responses: accumulation in the nucleus in response to alkaline but not sodium stress (PMID:17438333). Given the complexity already known from model yeast and other eukaryotes, positing simple linear models for C. albicans is not likely to optimize our understanding of the role of this important regulator. A linear model of signaling from Sak1 -> Snf1 -> Mig1 and Mig2 as in Fig. 7 clearly cannot adequately represent the findings. If the fungal cell required only linear signaling, presumably a two component system as in bacteria would suffice to respond to the availability of glucose. A recent paper comparing SNF1 signaling of Kluyveromyces lactis with that of S. cerevisiae shows how this might be done; they devised a less crude model of their findings (PMID: 26440109).

Our model only summarizes the new findings from our study. There is a recent review that summarizes the immense complexity of sugar sensing and signaling pathways in *Candida albicans* from Van Dijck (Front Microbiol. 2019; 10: 99.), mentioned above, for people who want a lot of detail. However, we did modify the summary figure (now Fig 8) to address one of the reviewer's earlier criticisms. We think that it is improved and we thank you for raising this issue.

I read the *K. lactis* paper that the reviewer recommends as a model for our paper. I agree that it is a very nice study. But I have to decline to use this paper as a model for this manuscript. The reason is that the paper has never been cited in PubMed or PMC, despite having been published over 4 years ago. Modeling our presentation on a never-cited paper does not seem to serve the interests of PLOS Genetics.

Minor issues:
1. A reviewer's work is made more time consuming by the absence of line numbering in the manuscript.

We sincerely apologize. Line numbers have been added to the newly revised manuscript.

2. "Carbon-source dependent transcriptional regulation of ScMIG1 and ScMIG2 expression has apparently been lost for C albicans MIG1 and MIG2." Unless the authors have evidence that the ancestral trait is carbon-source dependent transcriptional regulation of the MIG genes, while constitutive expression of these regulators is a derived trait, it is inappropriate to refer to such an evolutionary relationship. They

should simply state the observation.

We agree and the statement has been removed from the manuscript.

3. This reviewer finds the expression "up-" or "downregulated" to be somewhat inaccurate when describing the effect of mutations of transcriptional regulators on the expression of genes. Some of the effects of these mutations may be due to regulation, i.e. compensatory mechanisms of the cell to adapt to loss of a regulator; these effects are likely to be indirect. The most direct effects of the absence of these regulators, however, are aberrantly high or low expression levels of their target genes, i.e. elevated or diminished in comparison with the wild type.

The expressions "up-regulated" and "down-regulated" are terms used often in our field for this type of transcriptional analysis. We like them because they summarize observation without implying mechanism. We used motif analysis to discern likely direct targets, so the issue of direct vs indirect has been raised in the manuscript.

4. Setting up a hypothesis that mig1 mig2 mutants might be more fit during phagocytosis by macrophages seems a bit contrived and is not required to justify this experiment. It would be highly surprising if loss of 2 important transcriptional regulators were to increase fitness in a model host interaction. These phenotype experiments with the mutants (caspofungin sensitivity, biofilm formation, interaction with macrophages and endothelial cells) are of interest and do not need artificial justification.

The rationale takes up only 5 lines of manuscript, and it accurately represents our thinking. Although we agree that a pleiotropic mutation seems likely to cause a virulence defect, we note that there are many examples of pleiotropic mutations that cause no virulence defect (see Suzanne Noble's competition experiments) or even cause increased virulence in some assays. For example, increased virulence is seen for *C. albicans* null mutants of transcription factor genes *EFH1* and *PHO4*, for protein phosphatase gene *PPH3*, and even for topoisomerase gene *TOP2*. Therefore we wish to keep our brief rationale in place.

Have all data underlying the figures and results presented in the manuscript been provided?
Large-scale datasets should be made available via a public repository as described in the /PLOS Genetics/ data availability policy
<http://journals.plos.org/plosgenetics/s/data-availability>, and

numerical data that underlies graphs or summary statistics should be
provided in spreadsheet form as supporting information.

Reviewer #1: No: Transcriptomics data may still need to be deposited.

The RNA-sequencing data has been deposited at the SRA database and can be accessed using accession number for RNA-Seq data: PRJNA573104.

Reviewer #2: Yes

Reviewer #3: Yes

PLOS authors have the option to publish the peer review history of their article (what does this mean? <https://journals.plos.org/plosgenetics/s/editorial-and-peer-review-process#loc-peer-review-history>).
If published, this will include your full peer review and any attached files.

If you choose “no”, your identity will remain anonymous but your review may still be made public.

Do you want your identity to be public for this peer review? For information about this choice, including consent withdrawal, please see our Privacy Policy <https://www.plos.org/privacy-policy>.

Reviewer #1: No

Reviewer #2: No

Reviewer #3: No
