# Supplementary material for: Roles of Candida albicans Mig1 and Mig2 in glucose repression, pathogenicity traits, and SNF1 essentiality
Source: PLoS Genet. 2020 Jan 21;16(1):e1008582. doi: 10.1371/journal.pgen.1008582 (PMC6994163; doi:10.1371/journal.pgen.1008582)
Supplement: Attachment — Submitted filename: Response to reviews PGENETICS-D-19-01346R1 revision.pdf [file pgen.1008582.s017.pdf]

Comments to the Authors:
Please note here if the review is uploaded as an attachment.

Reviewer #1: The revised manuscript is a pleasure to read and represents a much more focused story that will be easier for readers to launch from. I have read through the paper and analyzed the new figures, and have no concerns.

I am so glad that the revision hit the mark!

The authors may consider a brief legend for supplemental tables to help with navigation within complex tables (e.g. Table S4).

This is an extremely helpful suggestion – thanks! We have added a tab to each complex supplemental table (S1-S6) called “Contents” or “Explanation” that introduces what the table describes and what the tabs contain.

Reviewer #2: Revision has dealt well with my concerns

Thank you!

Reviewer #3: The revised manuscript is improved and much clearer. Essentially it is a transcriptional analysis of the role of MIG1 and MIG2, which is worthwhile. It suggests that it is the inability to relieve otherwise constitutive transcriptional repression of Mig1 that makes snf1 mutants inviable.

I am so glad that we were able to frame the key findings suitably!

This reviewer is still somewhat uncomfortable with the manuscript’s manner in setting up results as surprises or answers to mysteries or puzzles e.g.
- essentiality of SNF1; the original Petter et al report in 1997 discussed the possibility of Snf1 possessing “some other unknown cellular functions” unrelated to carbon source adaptation not as an explanation for their inability to isolate homozygous null mutants in this gene, but because of the heterozygotes’ phenotypes of wild type carbon source utilization patterns together with a significant decrease in growth rate as well as morphological changes.

I regret to say that the reviewer is in error about what is said the in the paper by Petter et al. They discuss the possibility of a second Snf1 function in the last paragraph of the Discussion. The paragraph has 5 sentences with the following topics:
1. Sc Snf1 is not essential, though pleiotropic.
2. Barley Snf1 is required for growth and likely essential for development.
3. Ca Snf1 is essential.
4. Ca Snf1 may have additional essential regulatory roles.
5. Additional studies may reveal function of the essential regulatory component.

Please note that sentences 1-4 of the paragraph each contain the words “lethal” or “essential” or both; sentence 5 lacks those but includes the word “indispensable.” I am using the published words to draw the only simple conclusion, that the authors sought to explain Snf1 essentiality. The reviewer may have additional insight into what the authors were thinking, but that is not what the authors wrote. I submit that this misunderstanding provides strong justification for authors to write as clearly as possible what they think, and what the rationale may be for an

experiment. Such an approach should reduce the frequency of misunderstandings such as the one reflected in the reviewer's criticism

- the finding of decreased virulence behaviors of mig1 mig2 mutants in a macrophage model, when metabolic flexibility has been described by many authors as a critical virulence determinant in C. albicans (e.g. Ramirez-Zaval et al 2017). The rationale presented by the authors in their response is not convincing to this reviewer - 3 out of the 4 important genes whose mutants they cite as showing increased virulence have not been specifically studied for this phenotype to my knowledge, and the 4th, PHO4, has decreased virulence in several in vivo models.

This is a matter of taste more than anything else and just a suggestion to the authors that their readers may appreciate a measured presentation of rationales for experiments.

I agree that it is indeed a matter of taste, and we choose to retain the brief rationale that is in the first revision. Our goal is in part to help avoid misunderstandings, as discussed in the preceding rebuttal comment. Another important goal is to tell a story with a paper, not just publish a dataset or two.

Minor questions: why does the sak1 mutant appear twice in the heat map of Fig. 5?

We included the *sak1* mutant a second time, adjacent to the *snf1 mig* double/triple mutants to simplify visual comparison. We called attention of readers to this matter by stating, "The gene expression profile of a *snf1Δ/Δ* mutant cannot be assayed, so we used the *sak1Δ/Δ* strain for comparison to our *snf1Δ/Δ* strain panel (Fig 5 and S6 Table)."

Fig. 1B is mentioned in the text after Fig 2?

That is correct. However, importantly, we mention Figure 1 (specifically 1A) before Figure 2 is mentioned, so we followed journal guidelines.

We made Figure 1 and Figure 2 each a multi-panel figure, and each figure captures the analysis of a different dataset. Figure 1 captures glucose vs glycerol, while Figure 2 captures WT vs mig1 mig2. There is of course some conceptual overlap, as is the case in Figure 1B. We wrote the manuscript to present glucose vs glycerol first, then WT vs mig1 mig2. Therefore, it is only natural that we had to "back up" to Figure 1B after referring to Figure 2. As it stands, Figure 2 is pretty dense, so we sought to avoid putting another panel in it. In addition, it seemed unnecessarily complicated to introduce a separate figure for panel 1B. I hope that this strategy seems reasonable.

Have all data underlying the figures and results presented in the manuscript been provided?

Large-scale datasets should be made available via a public repository as described in the *PLOS Genetics* data availability policy, and numerical data that underlies graphs or summary statistics should be provided in spreadsheet form as supporting information.

Reviewer #1: Yes

Reviewer #2: Yes

Reviewer #3: Yes

PLOS authors have the option to publish the peer review history of their article (what does this mean?). If published, this will include your full peer review and any attached files.

If you choose “no”, your identity will remain anonymous but your review may still be made public.

Do you want your identity to be public for this peer review? For information about this choice, including consent withdrawal, please see our Privacy Policy.

Reviewer #1: No

Reviewer #2: No

Reviewer #3: No

In compliance with data protection regulations, you may request that we remove your personal registration details at any time. (Remove my information/details). Please contact the publication office if you have any questions.
